# Influence of Processing Parameters in Injection Molding on the Properties of Short Carbon and Glass Fiber Reinforced Polypropylene Composites

**DOI:** 10.3390/polym16192745

**Published:** 2024-09-28

**Authors:** Thomas Höftberger, Gernot Zitzenbacher, Christoph Burgstaller

**Affiliations:** 1 Transfercenter für Kunststofftechnik GmbH, Franz-Fritsch-Str. 11, 4600 Wels, Austria; thomas.hoeftberger@tckt.at; 2 School of Engineering, University of Applied Sciences Upper Austria, Stelzhamerstr. 23, 4600 Wels, Austria; gernot.zitzenbacher@fh-wels.at

**Keywords:** melt temperature, back pressure, pre-heating, tensile properties, fiber length

## Abstract

Short-fiber reinforcement is a potent approach to improving the material properties of injection-molded parts. The main consideration in such reinforced materials is to preserve the fiber length, as this is the major influence on the properties of a given composite. The aim of this work was to investigate the different influencing parameters in injection molding processing on the properties of short carbon and glass fiber-reinforced polypropylene. We investigated parameters like melt temperature and back pressure, but also machine size and pre-heating regarding their influence on the tensile properties. We found that adjustments of melt temperature and back pressure only yield small improvements in the fiber length and the tensile properties, also depending on machine size, but a pre-heating step of the granules can significantly improve the properties.

## 1. Introduction

Plastics are, due to their low weight and the low necessary forming temperatures, often the more sustainable alternative over other materials. As unreinforced polymers are often limited in strength and thermal behavior, reinforcing them with fibers is a widespread approach. Such materials are found in automotive applications, housing for power tools, or other technical parts. The performance of such reinforced materials depends on different parameters, e.g., fiber length and type, polymer matrix, and additives for promoting the force transfer between the matrix and the fibers [1,2,3].

While there are a lot of different options for fiber reinforcement, glass fibers are the most widespread due to their good balance between cost and achievable properties. Carbon fibers promise even higher reinforcement due to their high properties and are also thermally very stable but are prone to breaking due to their anisotropy originating from the production process [4]. To utilize the reinforcement potential of these fibers, processing needs to be as gentle as possible to preserve the fiber length. Usually, as a first step, mixing the fibers with the matrix is applied to produce a compound, and after that, a second step to produce a shape or part follows. In compounding, several investigations were conducted to find good processing conditions. We have reported on these as well as our own results elsewhere [5].

In injection molding, the material is conveyed, melted, and mixed by the screw, and in the injection stroke, the flow throws the gate into the mold cavity, where the fibers are further shortened, which influences the composite properties. The initial fiber length shows an influence, meaning that longer fibers usually stay longer when processed comparably [6]. Several researchers investigated the influence of the fiber length, fiber fraction, and to some limited extent of the processing parameters on the properties. One work investigates the influence of the fiber volume fraction of short carbon and glass fiber-reinforced composites. The authors found that with increasing fiber fraction, the average glass fiber length was reduced linearly with the fiber content, while for carbon fibers, the reduction in the mean fiber length increased with increasing fiber volume fraction in the composite, which is attributed to the higher fiber–fiber interaction as the carbon fibers are thinner and also due to the carbon fiber brittleness [7]. Another publication investigates the correlation between initial fiber length and achievable mechanical properties, where they found a direct correlation between the shorter fibers and the lower mechanical properties for the same fiber volume fraction [8]. Also, recycled carbon fibers were investigated for reinforcement. With these, dosing is usually an issue, as these are not neat short-chopped fiber bundles but irregular fiber agglomerates. In the referred work, the compatibilizer was used to embed the carbon fibers before further processing them into the compounds with the matrix polymer, thus enabling better processing in melt mixing so the recycled fibers can be utilized [9]. Another approach for utilizing such irregular fibers from composite recycling is to pre-compact them with a specialty pelletization to ensure the fibers can be properly dosed in a compounding unit [10]. Looking into the recycling of fiber-reinforced thermoplastics, the multiple processing steps reduce the fiber lengths and, therefore, the composite properties. As was shown in one work comparing glass, carbon, and natural fiber reinforcement, the fiber length reduction for carbon fibers is higher than for glass fibers (while natural fibers exhibit a different behavior as these also can fibrillate and are more flexible) due to the brittleness of the carbon fibers in multiple injection molding steps [11].

In regard to optimizing processing parameters, one work reports that low back pressure, as well as low injection velocity and screw speed, gives better mechanical properties of glass fiber-reinforced polypropylene [12]. Also, the presence of maleic anhydride-grafted polypropylene in carbon fiber composites with polypropylene is beneficial, as the load transfer is improved and thus the properties [13], while others investigated the fiber length influence specifically for short carbon fiber reinforcement [14].

As mentioned above, longer fibers improve the properties, so also specialty processing routes were investigated to reduce fiber breakage. One such route is to combine compounding and injection molding within one unit to reduce fiber length reduction [15]. Another approach is to directly feed fiber rovings into an injection molding machine, which helps to increase fiber length greatly, but here is the issue of residual agglomerates of fibers in the composite, as shear was too low to disperse the fibers properly [16].

Looking at the available data in the literature, what is missing is the investigation of the influence of different process parameters to optimize processing. While specialty equipment can give great results, such equipment is scarce and often expensive to purchase and operate. Therefore, we wanted to focus on the injection molding processing.

The aim of this work was to investigate the different influencing parameters in injection molding processing on the properties of short carbon and glass fiber-reinforced polypropylene to gain an understanding of the major influences and to deduce process settings that yield high-performing carbon fiber composites. The parameters investigated are melt and mold temperature and back pressure, as well as a pre-heating step. Also, injection molding machine size is considered by investigating the processing at three differently sized injection molding machines.

## 2. Materials and Methods

### 2.1. Materials and Formulations

In the first part of this work, we used 30 wt% reinforcement in polypropylene (grade HE125MO, Borealis, Linz, Austria) with a melt flow rate of 12 g/10 min at a temperature of 230 °C and 2.16 kg piston weight. To ensure proper compatibilization between the matrix and the fibers, 3 wt% of a maleic anhydride-grafted polypropylene (grade Scona TPPP8112 GA, BYK, Schkopau, Germany) with a graft level of about 1.4 wt% maleic anhydride and exhibiting an MFR value of about 80 g/10 min (at a temperature of 190 °C and 2.16 kg piston weight). As reinforcing fibers, 30 wt% of chopped glass fibers (HP3299 PPG, Pittsburgh, PA, USA) with a diameter of 14 µm and cut length of 4.5 mm or carbon fibers (PX35-55, Zoltek, Nyergesujfalu, Hungary) with a diameter of 7 µm and cut length of 6 mm were used. For the second set of investigations, the fiber content was reduced to 20 wt% glass or carbon fibers to reduce the fiber–fiber interaction. The maleic anhydride content was increased to 5 wt%, which leaves the remaining 75 wt% for the polypropylene matrix.

### 2.2. Processing

Compounds were produced using a co-rotating twin screw extruder (ThermoPrism TSE24HC, Thermo Fisher Scientific, Karlsruhe, Germany) with 24 mm diameter screws and a processing length of 40 L/D. All three components were dosed using a gravimetric dosing system. Polypropylene and coupling agent were dosed into the intake of the extruder, and fibers were dosed in Zone 8 (equal to 8 L/D residual processing length) via a side feeder unit, as reported elsewhere, together with the used screw geometry [5]. The throughput was kept constant at 10 kg/h with a screw rotational speed of 300 min^−1^. The extruded melt strands were cooled by a water bath, cut to 5 mm long granules by means of a strand cutter, and dried at a temperature of 90 °C for a duration of at least 1.5 h before injection molding processing.

For injection molding, three different machines were applied, respectively: an 80 t, 150 t, and 220 t machine. The smallest machine (Victory 80) exhibited 80 t clamping force and is equipped with a 3-zone screw with a 30 mm diameter. With this machine, different settings for melt and mold wall temperature as well as back pressure were investigated for molding the universal test specimen (geometry 1A according to ISO-527). The same specimen was also produced at a larger machine (Victory 150) with 150 t clamping force and a 3-zone screw with 35 mm diameter with varying the same parameters as above. Both machines are equipped with a non-return valve and a needle valve nozzle. The largest machine used in this work was a 220 t clamping force machine with a 55 mm diameter low compression screw, which conveys the melt with low shear. As tooling, a circular plaque with 2 mm wall thickness and the sprue in the center was molded, from which test specimens (geometry according to ISO-527 [17]) were cut in the radial direction by means of a mill (ICP 4030 XYZ, isel Austria GmbH, Wels, Austria). Variations in melt temperature and back pressure were investigated. All three injection molding machines were supplied by Engel, Schwertberg, Austria.

### 2.3. Materials Testing

Tensile testing was carried out according to ISO 527 on a Z020 universal test machine (Zwick/Roell, Ulm, Germany) equipped with pneumatic clamps with metal inserts with pyramid serrations, with a crosshead speed of 1 mm/min for determining the elastic modulus and 5 mm/min until the break of the samples. Five replicates were tested per sample. To retrieve the fibers from the different compounds, a macro-thermogravimetric analysis was performed using a Makro-TGA 701 device from Leco, Mönchengladbach, Germany. The fibers were kept for fiber length measurements after ashing the matrix at 450 °C.

As in this work, we always compare the results of testing within one set of test specimens; we did not further investigate the fiber orientation (morphology) of the samples, as due to the geometry and the same filling in injection molding, we do not expect any significant difference here.

Fiber length measurements were conducted using FASEP image analysis software (Version 1.90.44, IDM Systems, Darmstadt, Germany). The fibers were dispersed in deionized water (about 2 mg of fibers in 100 mL). Afterward, a petri dish was filled with this dispersion, and an image was taken using an Epson Perfection V800 scanner. This image was then processed using the FASEP software to evaluate the fiber length. In total, 1500 to 2500 fibers per sample were measured. The analysis of the values was carried out as described before [5] to yield the length-weighted fiber length, which is reported as the fiber length in this study.

## 3. Results and Discussion

### 3.1. Influence of Melt and Mold Wall Temperature

**Figure 1:**
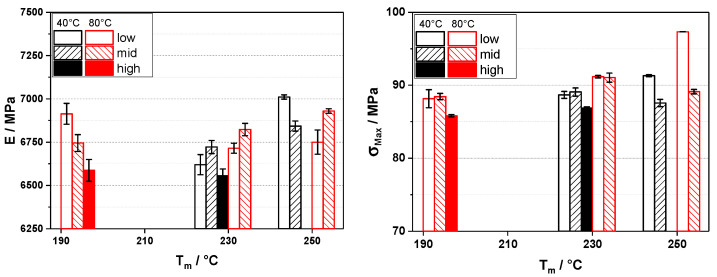
Elastic modulus (E, **left**) and tensile strength (σ_Max_, **right**) for 30 wt% glass fiber-reinforced polypropylene injection molded on an 80 t injection molding machine with different melt temperatures (T_m_), mold wall temperatures (40 and 80 °C), and back pressures (10, 100, and 250 bar for low, mid, and high, respectively).

**Figure 2:**
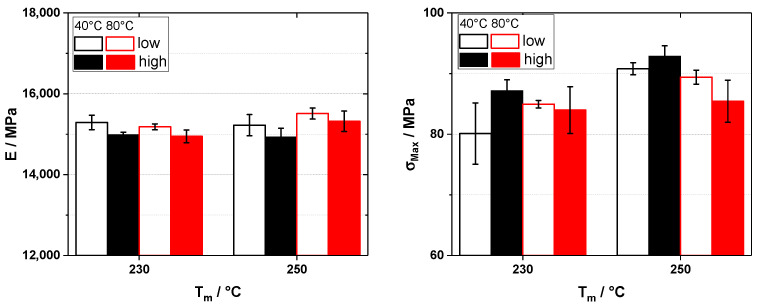
Elastic modulus (E, **left**) and tensile strength (σ_Max_, **right**) for 30 wt% carbon fiber-reinforced polypropylene injection molded on an 80 t injection molding machine with different melt temperatures (T_m_), mold wall temperatures (40 and 80 °C), and back pressures (10 and 150 bar for low and high, respectively).

In the first part of this work, we investigated the influence of the processing temperatures in injection molding on the achievable properties of carbon and glass fiber-reinforced polypropylene. As the reinforcing fibers show better composite properties the longer they are, preserving fiber length is the major goal here. A typical rule of thumb is to keep the temperatures high while keeping the back pressure low to reduce fiber breakage. In this way, melt viscosity is lower, and the forces acting on the fibers and shearing time can be held at a lower level. To test the validity of this rule, we varied the aforementioned parameters when injection molding universal test specimens. As shown in Figure 1, the elastic modulus of the glass fiber-reinforced material does not show a clear trend. At lower melt temperatures (190 °C), the lower back pressure seems to be beneficial, but this cannot be replicated at higher melt temperatures. In the case of the tensile strength, the trends are a bit more pronounced. With increasing melt temperatures, the tensile strength yields higher values, and also lower back pressures show a tendency to be beneficial. In addition, a higher mold wall temperature also yields higher values. In the case of the carbon fibers, as we know, these are more susceptible to breaking, as they are anisotropic in regard to the major fiber axis (i.e., carbon fibers take high loads in the direction of the main fiber axis but are less strong across it), so we replicated these experiments only with the higher levels of melt temperature. In Figure 2, the elastic modulus and tensile strength are plotted. Here, we see that for both properties, the settings do not seem to have any correlation. Reducing the back pressure to the minimum (10 bar) does only give marginal improvements, if any. In the case of the tensile strength, the higher melt temperature seems to be somewhat beneficial, but again, the improvement over the values for the specimen molded with a lower melt temperature is very limited.

### 3.2. Influence of Processing Step

To investigate where the major fiber length reduction is happening, so we can address that step specifically in our investigations, we ran an experiment where we took samples from the molded material after the melting in the machine, i.e., only extruded (without injection stroke) as well as dosed, meaning both times we caught the melt coming out of the machine on a metal plate without injecting it into the mold. Also, we have taken samples from the sprue, the runner, and the specimen. Furthermore, as we suspect the machine size (due to the difference in screw size and therefore a difference in applied shear) to be an influence, we conducted this experiment for an 80 t and a 150 t machine, which is equipped with a 30 and 35 mm screw, respectively. This set of experiments was conducted with 20 wt% of carbon fibers, as we chose the lower fiber content to reduce the fiber–fiber interaction for the rest of the investigations presented in this work.

**Figure 3:**
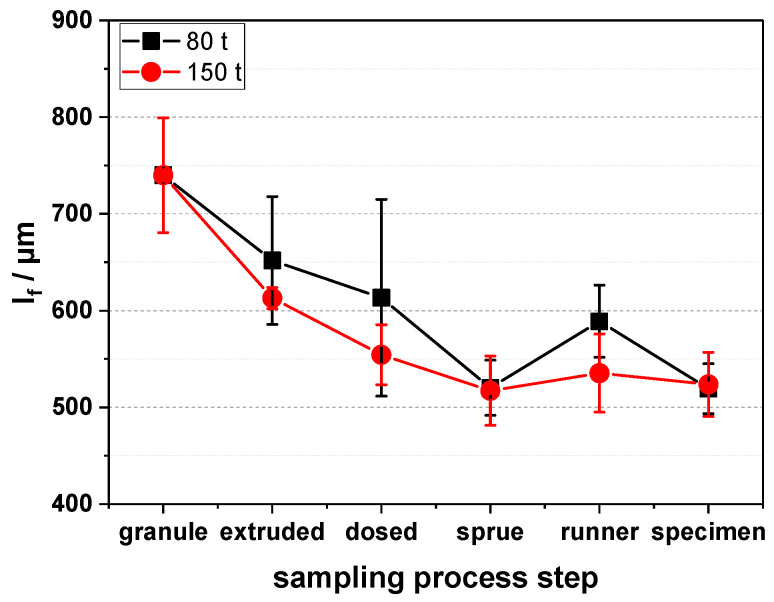
Fiber length (l_f_) vs. process step for 20 wt% carbon fiber reinforced polypropylene injection molded on an 80 t and 150 t injection molding machine.

As shown in Figure 3, the major fiber length reduction happens in the injection aggregate of the injection molding machine, where a fiber length reduction from about 750 µm to about 550–600 µm is found. When dosing the fibers without the injection stroke, we see less reduction than when the dosing, i.e., the injection stroke is happening. After that, the fiber length stays the same, regardless of whether the samples are taken from the sprue, the runner, or the specimen itself. This means that for preserving the fiber length, the melting and dosing step is the most crucial, as here, the most shear is applied to the material and, therefore, to the fibers. Also, the larger machine (150 t) does yield the same results, which could be due to the fact that the increase in screw diameter from 30 to 35 mm is not too high.

**Figure 4:**
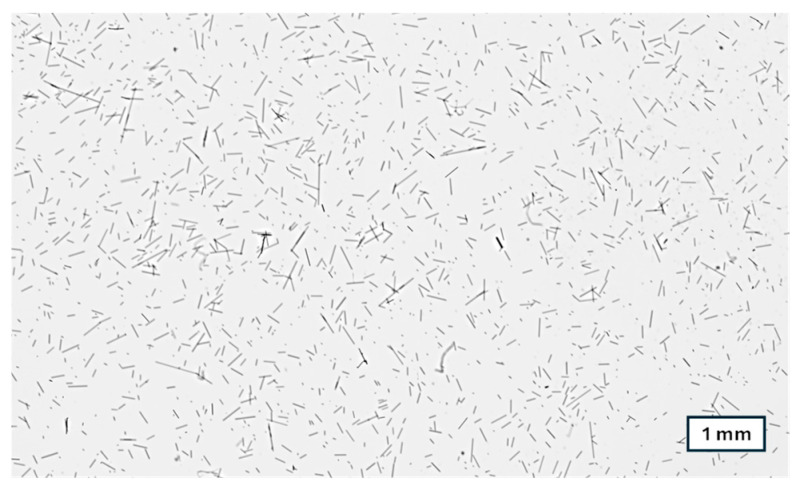
Micrograph from fiber length measurement of carbon fibers extracted from 20 wt% carbon fiber-reinforced polypropylene before injection molding processing.

Another aspect worth noting here is that the fiber length measurements exhibit a high level of scattering, as can be seen from the large error bars in Figure 3. One example of the scattering is that the fiber length in the runner seems to be higher than in the sprue for the 80 t machine, which is not possible but an effect of the fiber length measurements themselves. Although we use an automated length evaluation, there is still some influence of sample preparation (ashing the polypropylene to yield the fibers and dispersing them on a glass dish before the scanning), which can be seen from the large error bars. A higher number of samples for the measurement did not reduce the scattering. In our opinion, the scattering is also due to the fiber length distribution itself. A visual representation of this length distribution can be seen in Figure 4.

### 3.3. Influence of Machine Size

As the two machines used in the experiments are different in clamping force but do not differ that much in screw diameter, we wanted to investigate the influence of a larger machine (220 t clamping force, 55 mm screw diameter) on the achievable properties of carbon and glass fiber-reinforced polypropylene. As the bigger machine has much larger clamping plates than the smaller ones, it was not possible to utilize the universal test specimen tool. Instead, we used a circular plaque, which was injected from the center, and cut the specimen from the plaque. This results in lower values than for the universal test specimen due to the less oriented fibers in these cut specimens, which one has to keep in mind when comparing the absolute values with the results from above.

**Figure 5:**
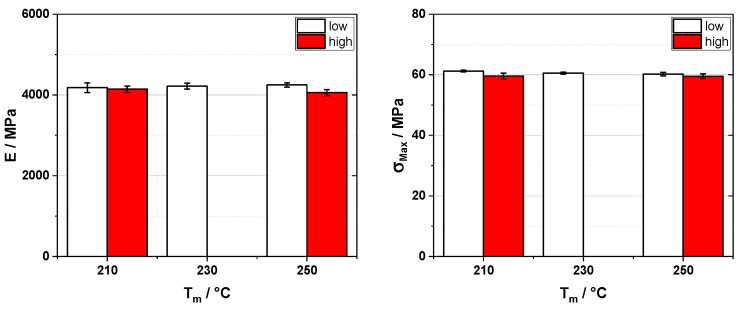
Elastic modulus (E, **left**) and tensile strength (σ_Max_, **right**) for 20 wt% glass fiber reinforced polypropylene injection molded on a 220 t injection molding machine with different melt temperatures (T_m_) and back pressures (10 and 100 bar for low and high, respectively).

Looking at the results for glass fiber-reinforced polypropylene, we see that in the case of the elastic modulus (Figure 5), the differences are negligible, as all the values of elastic modulus are scattered without a trend within 190 Mpa, or about 4% of the base value. In the case of the tensile strength, the differences are also low, but here, a trend can be seen that with higher back pressure, the tensile strength is slightly reduced. Another interesting trend can be seen when looking at the melt temperature T_m_, as here we do not see an improvement of the tensile strength with increasing melt temperature. This shows that at higher screw diameters, the influence of heating from the outside (i.e., the barrel) has less influence due to the low thermal conductivity of the polymer. Thus, less viscosity reduction due to higher barrel heating and, therefore, negligible influence on the mechanical properties is found.

**Figure 6:**
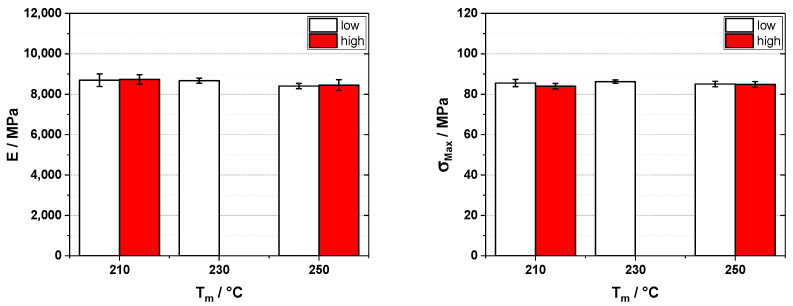
Elastic modulus (E, **left**) and tensile strength (σ_Max_, **right**) for 20 wt% carbon fiber-reinforced polypropylene injection molded on a 220 t injection molding machine with different melt temperatures (T_m_) and back pressures (10 and 700 bar for low and high, respectively).

For the carbon fiber-reinforced polypropylene (Figure 6), the same trends can be found. The values for the elastic modulus also lie within about 4% of the base value, and in the case of the tensile strength, a slight reduction can be found due to higher dwell pressures, but again, this is only a minor effect.

### 3.4. Alternative Strategies for Fiber Length Preservation

Due to the lack of success in preserving the fiber length and, therefore, the lack of improvement of the composite strength, we investigated alternative approaches in injection molding of short fiber-reinforced polypropylene. We are aware that successful approaches could be long fiber granules or alternative machine technologies, but as these are not widely available, we have not investigated them. Our approach is to set the processing parameters in a way that the shear and, therefore, damage to the fibers in the injection aggregate is as low as possible, especially for smaller machines and screw diameters, as we applied here for injection molding universal test specimens. Therefore, we investigated pre-heating the granules (by means of an additional drying hopper mounted on the intake of the 80 t injection molding machine) as well as an inverse temperature profile, i.e., having higher temperatures at the intake of the screw than on the die side. In detail, this means that the zones close to the intake are heated to 250 °C, while the die is held at 230 °C instead of the other way around. The intake zone itself needs to be cooled (40 °C) to avoid sticking the granules to the wall of the intake. The idea with the inverse temperature profile is to have lower shear and friction at the intake but not to overheat the polymer and, therefore, inflict too much damage.

As the carbon fibers are more prone to breakage, we focused in the last part on 20 wt% carbon fiber reinforced polypropylene, as these strategies can also be applied to glass fibers, due to them being more resistant to breaking.

**Figure 7:**
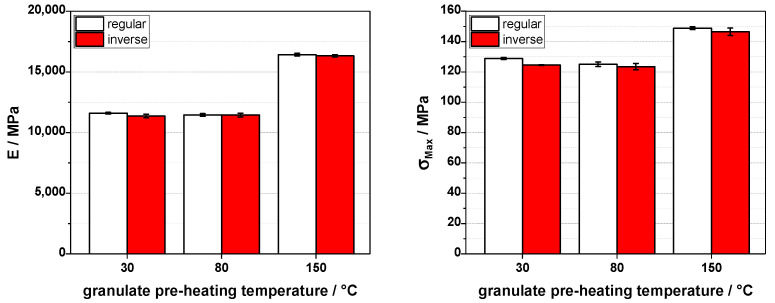
Elastic modulus (E, **left**) and tensile strength (σ_Max_, **right**) for 20 wt% carbon fiber-reinforced polypropylene injection molded on an 80 t injection molding machine with different granulate pre-heating temperatures and regular (230–250 °C from the intake to the die) and inverse (250–230 °C from the intake to the die) barrel temperature profiles.

As shown in Figure 7, the elastic modulus and the tensile strength increase with the pre-heating temperature, but only for the higher pre-heating temperature of 150 °C; the results are the same below. We suspect that behavior is due to the easier melting of the granules; therefore, less mechanical shear is applied to the material, which should keep the fibers longer. We do not see a gradual increase from room temperature (30 °C for the granules in the machine intake) over 80 °C to 150 °C, but a steep increase for the highest temperature can be explained by the thermal behavior of the matrix itself. As polypropylene is a semicrystalline material, temperature changes over the glass transition, but far below the melting point, show less influence due to the rigidity of the crystallites. When getting close to the melting point (about 160 °C in this case), we see a more pronounced effect of softening, as the material melting behavior is spread out over a region due to the melting of the differently sized crystallites in the polypropylene. This rather small range of softening is a well-known behavior of polypropylene, which also influences other processing routes, e.g., thermoforming proves to be more difficult than for amorphous polymers. Inverting the temperature profile along the injection molding screw does not yield a positive effect but gives a minor reduction in the elastic modulus and the tensile strength. This effect could be due to the higher shear at the end of the screw due to the constraint geometry (non-return valve and die) when in the inverse setting, the temperature is lower—especially at the smaller machine (80 t) we used here.

**Figure 8:**
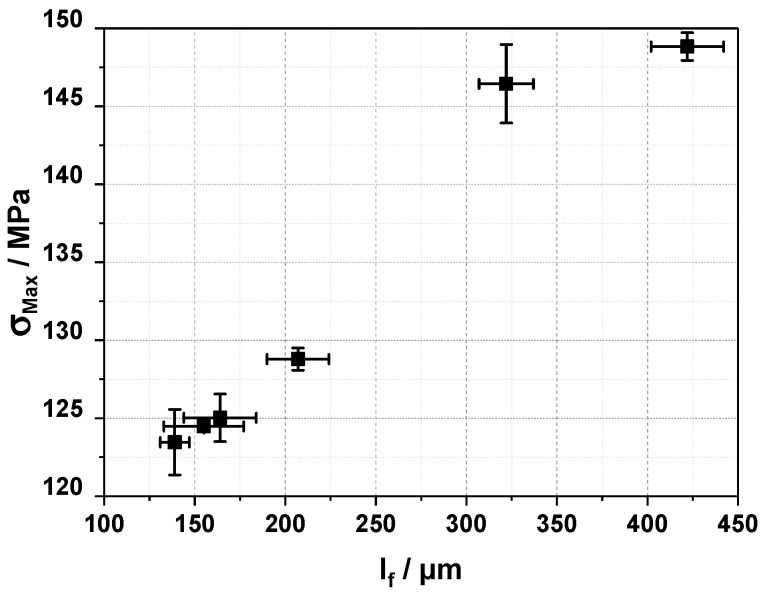
Tensile strength (s_Max_) vs. fiber length (l_f_) for 20 wt% carbon fiber reinforced polypropylene injection molded on an 80 t injection molding machine with different granulate pre-heating temperatures and regular (230–250 °C from the intake to the die) and inverse (250–230 °C from the intake to the die) barrel temperature profiles.

As the increase in the temperature is rather steep, we wanted to check if the fiber length correlates with that increase to make sure that there were no other effects overlooked here. In Figure 8, we plotted the fiber length vs. the tensile strength. One can see a nearly linear correlation between the two values, which lies well within the estimation one could make from fiber reinforcement theory [1] for subcritical fibers. We assume that we have subcritical fibers here due to the short fiber length and the high tensile strength, where a direct and linear increase with an increase in fiber length is found. Therefore, we can conclude that pre-heating at higher temperatures does have a positive effect on the fiber length and can be utilized to improve the composite properties.

## 4. Conclusions

In this work, we have investigated different approaches to improve the fiber length and, therefore, the composite properties of short carbon and glass fiber-reinforced polypropylene. We found that the regular rule of thumb approaches of reducing the back pressure as much as possible (from 250 to 10 bar) and increasing the melt temperatures (up to 250 °C) have limited effects on the achieved properties, being only within a few percent. For glass fiber-reinforced PP, we found a minor increase with these settings, but for carbon fiber-reinforced PP, we did not find any beneficial effect of these settings. Mold wall temperature did not show any positive effect in the investigated region. The reason why no or minor effects are shown due to the changes in melt temperature and back pressure is most likely due to the relatively small machines and their respective screw diameters applied in this work. We found comparable behavior for glass and carbon fibers, but due to their anisotropic nature, carbon fibers tend to break more easily, thus further limiting the effectiveness of the simple process parameter adaptions.

Another approach showed much more promising results—by increasing the temperature of the granules close to the melting point before feeding these into the injection molding machine, we achieved an increase of several 10% in elastic modulus and tensile strength, as the fiber length was preserved much better. Although this is also a process modification with a pre-heating hopper attached to the intake of the injection molding machine, this is a rather simple change, which can yield a large effect on the mechanical properties. Also, due to the fact that a lot of materials need to be pre-dried before injection molding anyway, this can be integrated for improved injection molding processing of short fiber reinforced thermoplastics.

## Data Availability

The original contributions presented in the study are included in the article, further inquiries can be directed to the corresponding author.
